# By downregulating Ku80, hsa-miR-526b suppresses non-small cell lung cancer

**DOI:** 10.18632/oncotarget.2808

**Published:** 2014-12-22

**Authors:** Zun-yi Zhang, Sheng-ling Fu, Su-qin Xu, Xiao Zhou, Xian-shen Liu, Yong-jian Xu, Jian-ping Zhao, Shuang Wei

**Affiliations:** ^1^ Department of Surgery, Tongji Hospital, Tongji Medical College Huazhong University of Science and Technology, Wuhan 430030, China; ^2^ Department of Respiratory and Critical Care Medicine, Tongji Hospital, Tongji Medical College Huazhong University of Science and Technology, Wuhan 430030, China

**Keywords:** hsa-miR-526b, Ku80, microRNA, NSCLC, p53

## Abstract

Ku80 is involved in DNA double-strand breaks (DSBs) repair. Ku80 is overexpressed in lung cancer tissues, yet, molecular mechanisms have not been examined. We identified that miRNA, hsa-miR-526b, is bound to the 3′-UTR of Ku80 mRNA, thus decreasing Ku80 expression in NSCLC cells. Hsa-miR-526b was downregulated in NSCLC tissues compared with corresponding non-tumorous tissues, and its expression was inversely correlated with Ku80 upregulation. Overexpression of Ku80 and downregulation of hsa-miR-526b were associated with poor clinical outcomes of NSCLC patients. Hsa-miR-526b suppressed NSCLC cell proliferation, clonogenicity, and induced cell cycle arrest and apoptosis. Hsa-miR-526b inhibited xenografts and orthotopic lung tumor growth. Further, Ku80 knockdown in NSCLC cells suppressed tumor properties *in vitro* and *in vivo* similar to hsa-miR-526b overexpression. In agreement, Ku80 restoration partially reversed cell cycle arrest and apoptosis induced by hsa-miR-526b in NSCLC cells *in vitro* and *in vivo*. In addition, hsa-miR-526b overexpression or Ku80 knockdown increased p53 and p21^CIP1/WAF1^ expression. These findings reveal that hsa-miR-526b is a potential target in cancer therapy.

## INTRODUCTION

Lung cancer is the leading type of cancer-related deaths worldwide, likely because it is often diagnosed at advanced stages that are beyond the optimal treatment period [1]. Non-small cell lung cancer (NSCLC) accounts for at least 80% of lung cancers [1]. The overall five-year survival rate for NSCLC in the United States remains as low as 16% when considering all stages and subtypes [2]. Despite advancements in treatments for NSCLC, including surgery, radiotherapy, chemotherapy, and recently developed molecular targeting therapies, such as inhibitors against epithelial growth factor receptor or vascular endothelial growth factor, the survival rates for the disease continue to be very low. Further investigation of the molecular mechanisms underlying tumorigenesis and progression of NSCLC is essential for developing new prognostic biomarkers and effective therapeutic targets.

Ku80 is well known for its critical function in repairing DNA double-strand breaks (DSBs) [3]. Misrepaired DSBs are major DNA lesions that can lead to chromosomal aberration, mutation, or carcinogenesis [4]. Previous studies have shown that overexpression of Ku80 may be associated with bladder cancer, cervical carcinoma, pancreatic cancer, and gastric cancer [5–7]. These studies provide evidence for a vital role of Ku80 in tumorigenesis. To date, little is known about the expression of Ku80 and its function in human NSCLC. In one study, a significant overexpression of Ku80 was observed in primary human lung adenocarcinoma, which was associated with poor clinical outcomes and resistance to cisplatin-based chemotherapy [8]. Despite growing evidence suggesting its role in cancer processes, the molecular mechanisms underlying aberrant Ku80 expression and its precise role in human NSCLC remain unexplored.

MicroRNAs (miRNAs) are a class of small noncoding RNAs that are approximately 22 nucleotides in length [9, 10]. MiRNAs can suppress posttranscriptional gene expression by binding to the 3′-untranslated regions (3′-UTRs) of mRNAs, effectively inhibiting translation or targeting mRNA degradation [11, 12]. MiRNAs are involved in cell proliferation [13], differentiation [14], and apoptosis [15]. Aberrant miRNA expression is associated with tumor formation and progression [16, 17]. A series of studies has shown that miRNAs can function as oncogenes or tumor suppressor genes in lung cancer. For example, miR-503 functions as a tumor suppressor gene targeting PI3K p85 and IKK-β in NSCLC, and its expression is inversely correlated with the clinical prognosis and overall survival of NSCLC patients [18]. The miR-17-92 cluster promotes cell proliferation and is overexpressed in lung cancer tissues [13]. MiR-198 inhibits proliferation and induces apoptosis of lung cancer cells via targeting FGFR1 [19]. MiR-545 suppresses cell proliferation, causes G_0_/G_1_ phase arrest, and induces cell apoptosis in lung cancer cells by targeting cyclin D1 and CDK4 genes [12]. MiR-31 and miR-143 may function to inhibit apoptosis in NSCLC by regulating ABCB9 and PKCε expression [20, 21]. Taken together, these reports indicate that miRNAs are involved in the development and progression of lung cancer, and that miRNAs represent a potential target in the treatment of lung cancer. However, the roles of dysregulated miRNAs in lung cancer are still not fully elucidated.

The purpose of this study was to identify miRNAs that regulate Ku80 expression in lung cancer and determine their functions. We examined the expression patterns of miRNAs in NSCLC tissues and evaluated the prognosis of patients with NSCLC. Our data revealed that hsa-miR-526b regulated NSCLC cell growth both *in vitro* and *in vivo* by directly targeting Ku80 gene. The potential mechanisms underlying growth regulation associated with hsa-miR-526b were also investigated.

## RESULTS

### Ku80 is significantly overexpressed in NSCLC tissues and is correlated with poor clinical outcomes

**Figure 1:**
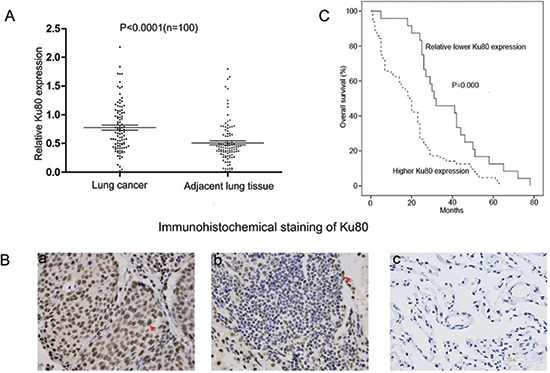
Ku80 is overexpressed in NSCLC tissues and its expression level is associated with lower survival rates **(A)** Quantitative analysis of Ku80 expression in 100 cases of paired NSCLC tissues and their corresponding adjacent lung tissues by western blot analysis. The P-value (*P* < 0.0001) corresponds to the comparison of Ku80 expression between the NSCLC tissues and corresponding adjacent lung tissues. **(B)** Representative immunostaining of Ku80 expression in human NSCLC tissues and corresponding adjacent lung tissues. (a) High levels of expression of Ku80 in NSCLC tissues, (b) low levels of expression of Ku80 in NSCLC, and (c) weak Ku80 staining in non-cancerous lung tissues. Arrows indicate positive nuclear staining for Ku80. **(C)** Kaplan–Meier analysis of overall survival in all NSCLC patients according to Ku80 protein level.

Ku80 expression was examined in 100 cases of paired NSCLC tissues and their corresponding adjacent lung tissues. A western blot analysis confirmed that Ku80 protein expression in 73 NSCLC samples was higher compared to their adjacent lung tissues (*P* < 0.0001; Fig. 1A and Supplementary Fig. S1). Immunohistochemical staining also indicated that a strong positive expression of nuclear Ku80 was frequently observed in NSCLC tissues, while very weak expression of Ku80 was found in most non-cancerous lung tissues (Fig. 1B). To further investigate whether the overexpressed Ku80 is correlated with the lower survival rates of NSCLC patients, we investigated an independent cohort of NSCLC patients. Kaplan-Meier analysis indicated that NSCLC patients with high Ku80 levels had a significantly shorter median overall survival compared to those with low Ku80 levels (*P* < 0.0001 by the log-rank test, Fig. 1C). Taken together, these data demonstrate that Ku80 was overexpressed in primary NSCLC tissue compared with normal lung tissue and high Ku80 levels were associated with lower survival rates in NSCLC patients.

In addition, the correlation between Ku80 expressions and clinicopathological parameters in the patients with NSCLC was assessed. Ku80 overexpression was significantly correlated with the tumor differentiation, smoking history, and elevated serum CEA level (*P* < 0.05, Table 1). However, there was no significant correlation between Ku80 expression and other clinicopathological parameters, such as age, sex, pathological pattern, lymphatic metastasis, tumor diameter, and tumor staging (*P* > 0.05, Table 1).

**Table 1:** The correlation between Ku80 upregulation and clincopathological parameters in the patients with NSCLC

Variables	Total cases (*n* = 100)	Ku80 expression level		*P* value^a^
		Upregulated (*n* = 73)	Intact (*n* = 27)	
**Age(years)**				0.264
≤ 50	21	17	4	
> 50	79	56	23	
**Gender**				0.19
Male	72	49	23	
Female	28	24	4	
**Differentiation**				0.001^b^
Low	39	35	4	
Moderate	38	29	9	
High	23	9	14	
**Smoking history**				0.037^b^
Never	34	29	5	
Ever	66	44	22	
**Pathological pattern**				0.098
SCC	54	38	16	
ADC	46	35	11	
**Lymphatic metastasis**				0.181
Yes	61	47	14	
No	39	26	13	
**Tumor diameter (mm)**				0.098
≤ 50	70	48	22	
> 50	30	25	5	
**CEA (ng/ml)**				0.009^b^
≤ 5	70	46	24	
> 5	30	27	3	
**Staging**				0.584
I-II	59	43	16	
III-IV	41	30	11	

a Chi-square test or the Fisher exact test;

b Statistically significant (*P* < 0.05)

### Hsa-miR-526b directly targets Ku80 in NSCLC cells

**Figure 2:**
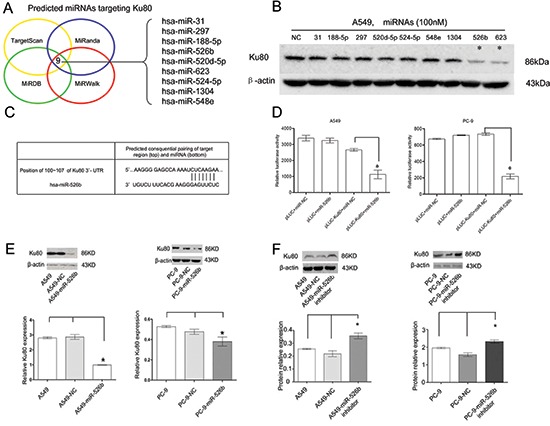
Hsa-miR-526b directly targets Ku80 in NSCLC cells **(A)** Venn diagram depicting the overlap between four miRNA databases that predict possible miRNAs targeting Ku80 gene, identifying nine potential miRNAs. **(B)** Western blot showing Ku80 expression levels in the nine miRNAs mimics and negative control RNA transfected A549 cells. β-actin was included as a loading control for each sample. **(C)** Predicted consequential pairing of Ku80 region and hsa-miR-526b by Targetscan. **(D)** Luciferase assays in A549 and PC-9 cells. pLUC-Ku80 vector was co-transfected with pre-miR-526b or pre-miR-NC. Relative suppression of luciferase expression was standardized to β-gal signal. Luciferase activity in pLUC-Ku80 group displayed a significant decrease following ectopic expression of hsa-miR-526b. **(E, F)** Ku80 protein was measured by western blot analyses 48 h after transfection. (E) Ku80 protein was downregulated in A549 and PC-9 cells transfected with hsa-miR-526b mimics. (F) Ku80 protein was upregulated in A549 and PC-9 cells with hsa-miR-526b inhibitor. *P < 0.05.

To determine whether miRNAs were involved in regulating Ku80 expression, we applied the four most commonly used databases of miRNA (TargetScan, MiRanda, MiRDB, and MiRWalk) to identify potential miRNAs that may target 3′-UTR of Ku80 mRNA. Nine miRNAs (hsa-miR-526b, hsa-miR-1304, hsa-miR-548e, hsa-miR-188-5p, hsa-miR-297, hsa-miR-524-5p, hsa-miR-31, hsa-miR-520d-5p and hsa-miR-623) were consistently identified by these four miRNA databases (Fig. 2A). Upon this determination, we transiently transfected these miRNAs mimics into A549 cell lines and evaluated Ku80 expression levels using a western blot analysis. Among the nine miRNAs, the downregulation of Ku80 by the hsa-miR-526b and hsa-miR-623 mimics was the most pronounced (Fig. 2B).

Subsequent luciferase assay indicated that hsa-miR-526b could directly target the binding site of Ku80 mRNA and suppress luciferase expression of pLUC-Ku80 (*P* < 0.01; Figs. 2C and 2D). When the control group was examined in a similar manner, hsa-miR-526b failed to demonstrate this effect (*P* > 0.05; Figs. 2C and 2D). Overexpression of hsa-miR-526b mimics in A549 and PC-9 NSCLC cell lines greatly reduced the protein level of Ku80 (*P* < 0.05; Fig. 2E). Conversely, inhibition of hsa-miR-526b increased Ku80 expression in both A549 and PC-9 NSCLC cell lines (*P* < 0.01; Fig. 2F). Taken together, our data show that hsa-miR-526b negatively modulated Ku80 expression by directly binding to its 3′-UTR.

### Hsa-miR-526b is downregulated in NSCLC tissues and its expression is associated with poor clinical outcomes

**Figure 3:**
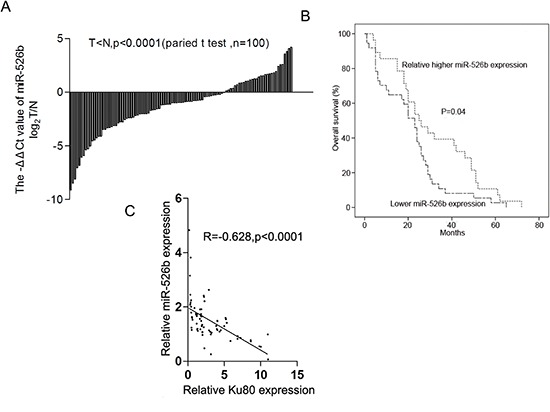
Hsa-miR-526b is downregulated in NSCLC tissues and its downregulation is inversely correlated with the Ku80 overexpression **(A)** Downregulation of hsa-miR-526b in NSCLC tissues compared with adjacent lung tissues (measured by TaqManqRT-PCR, *P* < 0.0001, paired t-test). **(B)** Kaplan–Meier analysis of overall survival in all NSCLC patients according to hsa-miR-526b expression level. **(C)** Downregulation of hsa-miR-526b is inversely correlated with Ku80 upregulation (*R* = −0.628, *P* < 0.0001, Pearson's correlation).

To further investigate its potential role, we evaluated the expression levels of hsa-miR-526b in 100 human NSCLC tissues and their corresponding adjacent lung tissues using qRT-PCR. Interestingly, hsa-miR-526b was significantly downregulated in 69% NSCLC tissues compared with adjacent lung tissues (*P* < 0.0001 by paired *t*-test; Fig. 3A). Kaplan-Meier log-rank analysis also indicated that NSCLC patients with low hsa-miR-526b level had a significantly shorter median overall survival compared to those with high hsa-miR-526b expression (*P* = 0.04; Fig. 3B). Moreover, the abundance of hsa-miR-526b was inversely correlated with that of Ku80 in NSCLC tissues (*r* = −0.6281, *P* < 0.0001; Fig. 3C). Next we examined the correlation between hsa-miR-526b expression level and the clinicopathological parameters in patients with NSCLC. Hsa-miR-526b downregulation was significantly correlated with tumor differentiation, smoking history, tumor diameter, and elevated serum CEA level (*P* < 0.05; Table 2). No correlation was found between miR-526b expression and other clinicopathological parameters in patients with NSCLC (*P* > 0.05; Table 2).

**Table 2:** The correlation between hsa-miR-526b downregulation and clincopathological parameters in the patients with NSCLC

Variables	Total cases (*n* = 100)	miR-526b expression level		*P* value^a^
		Down regulated (*n* = 69)	Intact (*n* = 31)	
**Age (years)**				0.494
≤ 50	21	14	7	
> 50	79	55	24	
**Gender**				0.06
Male	72	46	26	
Female	28	23	5	
**Differentiation**				0.001^b^
Low	39	34	5	
Moderate	38	25	13	
High	23	10	13	
**Smoking history**				0.013^b^
Never	34	29	5	
Ever	66	40	26	
**Pathological pattern**				0.57
SCC	53	35	18	
ADC	47	34	13	
**Lymphatic metastasis**				0.197
Yes	61	45	16	
No	39	24	15	
**Tumor diameter(mm)**				0.000^b^
≤ 50	70	42	28	
> 50	30	27	3	
**CEA(ng/ml)**				0.000^b^
≤ 5	70	41	29	
> 5	30	28	2	
**Staging**				0.585
I	14	8	6	
II	45	32	13	
III-IV	41	29	12	

a Chi-square test or the Fisher exact test;

b Statistically significant (*P* < 0.05)

### Hsa-miR-526b suppresses cell growth and colony formation in NSCLC cells

**Figure 4:**
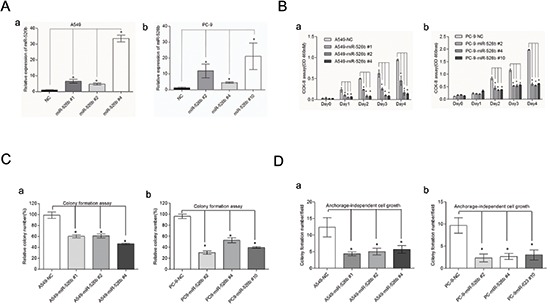
Hsa-miR-526b demonstrates a tumor suppressor role in NSCLC **(A)** Relative expression of hsa-miR-526b detected by TaqManqRT–PCR in two NSCLC cell lines stably transfected with hsa-miR-526b or hsa-miR-NC. **(B)** Hsa-miR-526b overexpression significantly suppressed the proliferation of A549 and PC-9 cells by using CCK-8 assay. Overexpression of miR-526b decreased cell clonogenicity in both A549 and PC-9 cells. **(C)** Histograms indicated that hsa-miR-526b can markedly inhibit the plate colony formation in A549 and PC-9 cells. **(D)** Histograms showing significant suppression of anchorage-independent cell growth in A549 and PC-9 cells by hsa-miR-526b. The results shown represent the mean ± SD of triplicate experiments. *P < 0.05 between groups of cells.

To study the effects of hsa-miR-526b on cell growth, hsa-miR-526b expressing plasmid and its negative control plasmid (NC) were transiently transfected into two NSCLC cell lines, A549 and PC-9, respectively. Hsa-miR-526b-expressing and NC-transfected stable A549 and PC-9 cell clones were generated. The qRT-PCR analysis confirmed that hsa-miR-526b stably expressing A549 cell clones (#1, #2, and #4) and PC-9 cell clones (#2, #4, and #10) expressed much higher levels of hsa-miR-526b, whereas the clones with the NC showed very low level of hsa-miR-526b expression (*P* < 0.05; Fig. 4A). Western blot analysis also indicated that hsa-miR-526b stably expressing A549 and PC-9 cells expressed much lower protein levels of Ku80 than those of control cells (Supplementary Fig. S2). Hsa-miR-526b overexpressing A549 and PC-9 stable clone cells grew at significantly slower rates compared with those of the NC-transfected cells (*P* < 0.01; Fig. 4B). Furthermore, to assess the functional role of hsa-miR-526b in tumor formation, the plate colony formation and anchorage-independent growth were measured in A549 and PC-9 cells. Analysis of clonogenicity showed that hsa-miR-526b overexpressing stable clone cells displayed much fewer and smaller colonies than NC-transfected cells (Supplementary Fig. S3). Analysis of colony formation and cell growth showed that there was a significant difference among the different cell groups (*P* < 0.05; Figs. 4C and 4D).

### Hsa-miR-526b inhibits cell cycle progression and induces apoptosis in NSCLC cells

**Figure 5:**
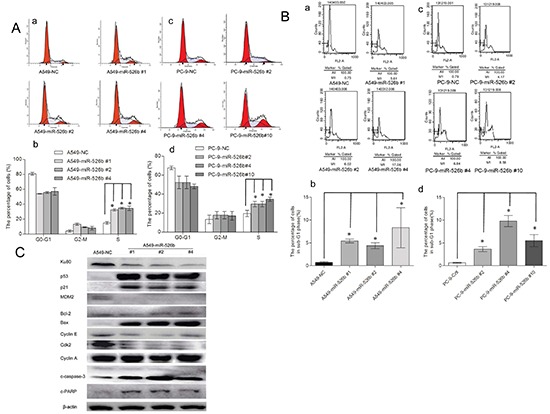
Hsa-miR-526b induces cell cycle arrest and apoptosis **(A)** Distribution of cells in G0/G1, S, and G2/M phases is shown for miR-526b or NC stably transfected-A549 and PC-9 cells. Cell cycle was determined by FACS. A statistically significant increase in S-phase percentage was observed. *P < 0.01. **(B)** Overexpression of hsa-miR-526b induced apoptosis: miR-526b or NC stably transfected-A549 and PC-9 cells were assessed by flow cytometric analysis. The percentage of apoptotic cells is shown as subG1 fraction. One representative experiment out of three is shown. A statistically significant increase in apoptotic rate was observed. *P < 0.01. **(C)** Western blot showing the expression levels of Ku80, p53, p21, MDM2, cyclinA, cyclinE, cdk2, Bax, Bcl-2, c-caspase-3 and c-PARP in hsa-miR-526b-expressing cells and control cells. β-actin was included as a loading control for each sample. The results are expressed as the means ± SD of three separate experiments.

To explore the underlying mechanism of the cell growth suppression caused by hsa-miR-526b overexpression, the cell cycle distributions of the hsa-miR-526b expressing and the NC-transfected A549 and PC-9 cells were analyzed. Flow cytometry analysis demonstrated increased numbers of cells from the hsa-miR-526b overexpressing A549 or PC-9 cell clones in S-phase compared with those in the NC-transfected clones (*P* < 0.05, Fig. 5A). These data show that reinforcement of hsa-miR-526b expression inhibits cell cycle progression, which may be S-phase arrest.

The subG_1_ phase refers to cells that have less DNA content than normal cells, indicating apoptosis. Our data indicated that the percentage of subG1 in the NC-transfected A549 cells, miR-526 #1, miR-526 #2, and miR-526 #4 clone cells were 0.73 ± 0.26%, 5.42 ± 0.72%, 5.38 ± 0.83%, and 13.69 ± 3.24%, respectively (Fig. 5B(a)). There was a significant difference in cell apoptotic rate between hsa-miR-526b expressing clone cells and the NC-transfected A549 cells (*P* < 0.01, Fig. 5B (b)). The subG_1_ percentage in the NC-transfected PC-9 cells and the miR-526 #2, miR-526 #4, and miR-526 #10 clone cells were 0.65 ± 0.20%, 3.64 ± 0.76%, 9.79 ± 1.71%, and 5.5 ± 1.88%, respectively (Fig. 5B (c)). There was a significant difference in cell apoptotic rate between hsa-miR-526b expressing clone cells and the NC-transfected PC-9 cells (*P* < 0.01, Fig. 5B (d)). These data indicate that apoptosis is also involved in the miR-526-induced cell growth inhibition.

The expressions of S-phase and cell apoptosis associated molecules were further investigated (Fig. 5C). The expression levels of p53 and p21^Cip1/Waf1^ were increased in the hsa-miR-526b expressing cells compared with those of NC-transfected A549 cells (*P* < 0.05), whereas the expression levels of MDM2, cdk2, and cyclinE were decreased (*P* < 0.05). The expression level of cyclinA remained unchanged (*P* > 0.05). The ratio of Bax/Bcl-2 expression levels was increased in the hsa-miR-526b expressing cells (*P* < 0.05). In addition, as apoptotic markers, the expression levels of c-PARP and c-caspase-3 were also increased in the hsa-miR-526b expressing cells compared with those of NC-transfected A549 cells (*P* < 0.05).

### Hsa-miR-526b inhibits xenografts and orthotopiclung cancer growth in nude mice

**Figure 6:**
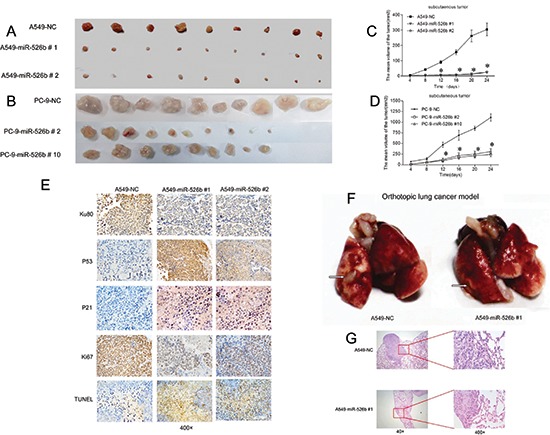
Hsa-miR-526b inhibits xenografts and orthotopic lung cancer growth in nude mice The subcutaneous tumors derived from the hsa-miR-526b overexpressing clones were smaller in size than those from the NC-transfected clones in A549 **(A)** and PC-9 cells **(B)**. Tumor growth curves showed that the tumors derived from the hsa-miR-526b overexpressing A549 **(C)** or PC-9 **(D)** clone cells grew significantly slower than those from the control cells at all time points past 12 days after injection. *P < 0.01. The mean and SD for tumor volumes were determined for each group. **(E)** The expression status of Ku80, p53, p21, and Ki67 in the xenograft tumor tissues. **(F)** The orthotopic tumor implantation assays demonstrated that the lung tumors (left) derived from A549-miR-526b 1# cells were markedly smaller in size compared with those from NC-transfected A549 cells 30 days after orthotopic injection. Arrows: visible tumor on the lung surface. **(G)** Representative H&E staining of hsa-miR-526b and NC-transfected A549 cells with formed orthotopic lung tumor tissues.

Sixty 4-week old male athymic nu/nu mice were divided into six groups (*n* = 9–10/group). The mice received 3 × 10^6^ cells of miR-526b expressing clones and the NC-transfected A549 or PC-9 clone cells, respectively, by subcutaneous injection into the flank. Tumor volumes were measured with calipers every four days after injection. The tumors were removed from the sacrificed mice on day 24 after injection. As shown in Figs. 6A and 6B, the mean volumes of the tumors derived from the miR-526 expressing clones were significantly smaller than those derived from the NC-transfected clone cells both in A549 and PC-9 (*P* < 0.01). In addition, tumors derived from the miR-526b-expressing clone cells (both in A549 and PC-9 cell) grew at significantly slower rates at all time points examined since day 12 after injection relative to the control groups (*P* < 0.01; Figs. 6C and 6D). Immunohistochemical staining study indicated that p53 and p21^Cip1/Waf1^ expressions were increased in the tumor tissues derived from the miR-526b-expressing cells compared with those in the tumors from the NC-transfected A549 cells (*P* < 0.01; Fig. 6E), while expression of Ku80 and Ki67 was decreased (*P* < 0.01; Fig. 6E). TUNEL assay indicated that apoptotic rate in the tumor tissues derived from the hsa-miR-526b expressing cells was markedly higher than that from the NC-transfected A549 cells (*P* < 0.05; Fig. 6E). The alteration pattern of these protein expressions in xenograft tumor tissues was consistent with that of those protein expressions identified *in vitro* in miR-526b-expressing cells and NC-transfected cells.

Due to well mimicking the real tumor growth environment [22], we further established an orthotopic tumor nude mice model to evaluate the effect of hsa-miR-526b on the lung cancer growth. Our data demonstrated that the volume of orthotopic lung tumors derived from A549-miR-526b #1 cells was significantly smaller compared with that of the tumors derived from NC-transfected A549 cells (*P* < 0.05; Figs. 6F and 6G).

### Hsa-miR-526b suppresses NSCLC cell growth *in vitro* and *in vivo* by downregulating Ku80 expression

**Figure 7:**
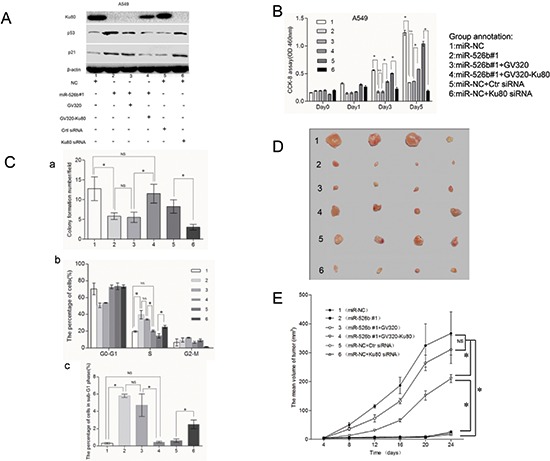
Hsa-miR-526b functions as a tumor suppressor in lung cancer by targeting Ku80 via p53/p21 participation **(A)** Western blot showing the expression levels of Ku80, p53, and p21 in A549-NC cells, A549-miR-526b 1# cells, and A549-miR-526b 1# cells transfected with GV320-Ku80 or GV320, and A549-NC cells transfected with Ku80 siRNA or control siRNA. **(B)** Cell proliferation detected in different cell groups at 0d, 1d, 3d and 5d after transfection. **(C)** (a) Histogram illustrating the relative colony numbers per field of the indicated cell groups. (b) Histogram showing the percentage of cells in G0-G1, S and G2-M cell-cycle phases. (c) Histogram showing the apoptotic percentage (shown as subG1 fraction) of the indicated cell groups. **(D)** The subcutaneous tumors derived from indicated cell groups (*n* = 4 each). **(E)** Tumor growth curves demonstrating that transfection of GV320-Ku80 in A549-miR-526b #1 cells significantly promoted subcutaneous tumor growth, while intratumoral injection of cholesterol-conjugated Ku80 siRNA significantly suppressed subcutaneous tumor growth compared to control groups. The mean and SD for tumor volumes were determined for each group. NS, *P* > 0.05; *P < 0.05.

To confirm the involvement of Ku80 in the anti-tumor effects of hsa-miR-526b, we transfected Ku80-expressing lentiviral vector (GV320-Ku80) and empty lentiviral vector (GV320) into A549-miR-526b #1 cells for further experiments (Fig. 7A). After GV320-Ku80 was introduced into the A549-miR-526b #1 cells, suppression of cell proliferation and clonogenicity, as well as induction of cell cycle arrest and apoptosis by hsa-miR-526b, was reversed to a certain extent (Figs. 7B and 7C and Supplementary Fig. S4). Relative to the control group, overexpression of Ku80 in A549-miR-526b #1 cells significantly promoted subcutaneous tumor growth *in vivo* (*P* < 0.05; Figs. 7D and 7E). These results suggested that Ku80 could partially overcome the growth suppression, cell cycle arrest, and apoptosis induction of hsa-miR-526b in NSCLC cells *in vitro* and *in vivo*.

To further identify the role of Ku80 in NSCLC pathogenesis, we examined whether inhibition of Ku80 resulted in repression of NSCLC, similar to that observed with miR-526b overexpression. Ku80 siRNA and control siRNA were introduced into NC-transfected A549 cells (Fig. 7A). We observed that specific knockdown of Ku80 by siRNA could result in the suppression of cell proliferation and clonogenicity, as well as induction of cell cycle arrest and apoptosis, similar to that seen in A549-miR-526b #1 cells (Figs. 7B and 7C and Supplementary Fig. S4).

In the nude mouse model, compared with control group, intra-tumor injection of cholesterol-conjugated Ku80 siRNA inhibited Ku80 expression and significantly suppressed subcutaneous tumor growth (*P* < 0.05; Figs. 7D and 7E). In summary, these data suggest that negative regulation of Ku80 contributed to the anti-tumor effects of hsa-miR-526b involved in NSCLC.

### P53/p21 pathway may be involved in the anti-tumor effect of hsa-miR-526b by targeting Ku80

Previous studies have demonstrated a close relationship between Ku80 and p53/p21 signaling [23, 24]. The role of the p53/p21 signaling pathway in the regulation of cell cycle progression and apoptosis is well established [25]. Therefore, we investigated whether the p53/p21 pathway was involved in the anti-tumor effect of hsa-miR-526b. We found that overexpression of hsa-miR-526b or knockdown of Ku80 in A549 cells significantly enhanced the expression of the p53 and p21^Cip1/Waf1^ (*P* < 0.05; Fig. 7A). In addition, the immunohistochemical staining study indicated that p53 and p21^Cip1/Waf1^ expression was increased in the tumors derived from the A549-miR-526b #1 cells compared with those in the tumors from the NC-transfected cells (Fig. 6E). Moreover, restoration of Ku80 expression also significantly inhibited the expression level of p53 and p21^Cip1/Waf1^ in A549-miR-526b #1 cells (*P* < 0.05; Fig. 7A). Thus, p53/p21 pathway may play an important role in the anti-tumor effects of hsa-miR-526b by targeting Ku80 in NSCLC.

## DISCUSSION

Ku80 is involved in multiple cellular processes, such as telomere maintenance, gene transcription, chemotherapy and radiotherapy resistance, cell growth, and apoptosis [4, 26, 27]. In the present study, we demonstrated that Ku80 was significantly overexpressed in NSCLC tissues compared with that in adjacent lung tissues, and this overexpression was significantly correlated with the survival of NSCLC patients. These results are in agreement with those recently reported [8, 28]. Our results also suggested that Ku80 overexpression was significantly correlated with tumor differentiation, smoking history, tumor diameter, and serum CEA level in patients with NSCLC. However, a previous study found that overexpression of Ku80 was correlated with tumor staging and lymphnode metastasis status in patients with lung adenocarcinoma [8]. The causes leading to the inconsistent results may be due to the inclusion of more pathological types of lung cancer than that of the previous study. We also demonstrated that Ku80 knockdown by RNA interference significantly suppressed the NSCLC cell proliferation, caused cell-cycle arrest, and cell apoptosis. This result was consistent with a previous study showing that knockdown of Ku80 decreased the viability of A549/DDP cells and promoted cisplatin-induced apoptosis [8]. In summary, we demonstrated that Ku80 was highly expressed in NSCLC and knockdown of Ku80 significantly inhibited cell proliferation by inducing cell cycle arrest and apoptosis.

Many studies have shown that miRNAs can function as oncogenes or tumor suppressors by post-transcriptionally inhibiting numerous target gene expressions in lung cancer [29–31]. In order to investigate whether miRNAs were involved in the mechanism of Ku80 dysregulation in NSCLC, we used four different miRNA databases to predict which miRNAs target Ku80. We successfully identified two miRNAs (hsa-miR-623 and hsa-miR-526b) that could significantly inhibit Ku80 expression in A549 cells. Our study indicated there was significantly different function between hsa-miR-623 and hsa-miR-526b in NSCLC (data not shown), so we chose to investigate the role of hsa-miR-526b in this paper. Until now, investigation into the role of hsa-miR-526b has been very limited. A recent genome-wide association study found that the hsa-miR-526b binding-site rs8506G > A polymorphism in the lincRNA-NR_024015 exon predisposes Chinese people to a higher risk of non-cardiac gastric cancer [32]. Hsa-miR-526b is identified as one of the pregnancy-associated miRNAs found in maternal plasma [33]. As far as we know, the expression patterns of hsa-miR-526b and its role in human cancer, including human NSCLC are unknown. The present data demonstrated that hsa-miR-526b directly bound to the 3′-UTR of Ku80 mRNA and markedly inhibited Ku80 expression. Hsa-miR-526b was found to be frequently down-regulated in NSCLC tissues when compared with adjacent non-cancerous tissues, and it showed an inverse significant correlation with Ku80 expression. Clinicopathological correlation analysis showed that hsa-miR-526b expression was significantly correlated with tumor differentiation, smoking history, tumor diameter, and serum CEA level in patients with NSCLC, which was similar to the results found in the study of Ku80. Further study indicated that hsa-miR-526b significantly suppressed lung cancer cell proliferation *in vitro*. Hsa-miR-526b was shown to significantly inhibit tumor growth both in the subcutaneous xenografts and orthotopic lung cancer nude mouse model. Taken together, these data suggest that hsa-miR-526b functioned as a tumor suppressor in NSCLC. To the best of our knowledge, this is the first attempt to illuminate the expression pattern of hsa-miR-526b and its role in NSCLC using both *in vitro* and *in vivo* models.

Further studies were performed to illustrate the molecular mechanisms of anti-tumor effect of hsa-miR-526b in NSCLC. Our study showed that Ku80 was upregulated in NSCLC tissues, and an inverse correlation was observed between hsa-miR-526b and Ku80 expression in NSCLC tissues. In addition, silencing of Ku80 by RNAi inhibited tumor properties of lung cancer cells in a manner resembling that of hsa-miR-526b overexpression. Moreover, restoration of Ku80 could partially reverse the anti-tumor effects induced by hsa-miR-526b both *in vitro* and *in vivo*. Thus, downregulation of Ku80 may, at least partially, explain the anti-tumor effects of hsa-miR-526b in NSCLC. Silencing Ku80 by RNAi inhibited the proliferation in lung cancer cells similar to the phenomenon observed in cervical carcinoma and esophageal squamous carcinoma cells [34, 35]. Molecular mechanisms contributing to NSCLC pathogenesis have been extensively investigated. Mutant vital genes and dysregulating cell signaling pathways, including EGFR [27, 36], HER_2_ [37], EML_4_-ALK [38], RAS/MEK [39], PI3K/Akt/mTOR [40], and P53/P21 [41–43] have been found to play pivotal roles in NSCLC carcinogenesis and progression. Among them, the activated p53/p21 signal is well established as a critical determinant in controlling both cell-cycle arrest and apoptosis [25, 44]. The p53/p21^Cip1/Waf1^-dependent checkpoint control of G1/S and G2/M phases of the cell cycle in response to DNA damage is an important mechanism of genome stability maintenance in normal cells [45]. In our study, overexpression of hsa-miR-526b or knockdown of Ku80 was shown to induce S-phase cell cycle arrest and apoptosis. Overexpression of hsa-miR-526b or knockdown of Ku80 in A549 cells significantly enhanced the expression levels of the p53 and p21^Cip1/Waf1^, while restoration of Ku80 expression also significantly inhibited p53 and p21^Cip1/Waf1^ expression in hsa-miR-526b expressing A549 cells. These findings suggest that the anti-tumor effect of hsa-miR-526b may be mediated by p53/p21 pathway. Consistent with our results, Li et al. reported that functional inactivation of the second Ku80 allele (heterozygous for Ku80) in HCT116 cells resulted in induction of the tumor suppressor protein p53, which may contribute to the inhibition of cell growth and induction of apoptosis [46]. P53 and p21^Cip1/Waf1^ were also found to be increased in *Ku80^−/−^* murine embryonic fibroblasts, and *Ku80* deletion ameliorated tumor burden in *APC^MIN^* mice [23]. Indeed, a single miRNA has been thought to target multiple mRNAs and regulate gene expressions [47]. Therefore, there may be other molecules or signaling pathways which are also targeted by hsa-miR-526b, and some of them may be still unknown in NSCLC. This presumption may guide future studies to determine the functions of hsa-miR-526b in NSCLC carcinogenesis and progression.

Taken together, our results demonstrate that hsa-miR-526b directly targets Ku80, which initiates apoptosis and S-phase arrest via p53-p21 participation during human lung cancer cell proliferation. Moreover, overexpression of hsa-miR-526b or knockdown of Ku80 significantly suppresses the NSCLC growth *in vitro* and *in vivo*. Our studies provide a rationale for the development of hsa-miR-526b or Ku80 as a potential therapeutic target against NSCLC.

## MATERIALS AND METHODS

### Antibodies and reagents

Ku80 siRNA used for *in vitro* transfection and *in vivo* cholesterol-conjugated Ku80 siRNA delivery, hsa-miR-526b mimics and inhibitors used for *in vitro* transfections, and their respective negative controls (NC) were from Ribobio Co. (Guangzhou, China). The hsa-miR-526b-expressing plasmid, GV268-miR526b, the Ku80-expressing lentiviral vector, GV320-Ku80, and their corresponding control vectors were purchased from Genechem (Shanghai, China). Anti-Ku80 (Ab-2), p21^Cip1/Waf1^ (F-5), p53 (Do-1), cleaved-PARP (194C1439), cleaved-caspase-3 (h176), cdk2 (M2), cyclin E (C-19), cyclin A (M20), MDM2 (SMP14) and β-actin (C4) antibodies were purchased from Santa Cruz Biotechnology (USA).

### Patients and specimens

One-hundred pairs of human NSCLC tissues and their corresponding adjacent lung samples were obtained from patients who underwent lung resection surgery at the Thoracic Surgery Center of Tongji Hospital affiliated with Huazhong University of Science and Technology between 2007 and 2010. NSCLC tissues were confirmed pathologically, and all specimens were stored at −80°C until used for analysis. Clinicopathologic histories for these patients including age, gender, differentiation, smoking history, pathological pattern, lymphatic metastasis, tumor diameter, serum CEA level, and tumor staging are shown in Tables 1 and 2. NSCLC was staged according to the tumor-node-metastasis (TNM) staging system of the Union for International Cancer Control (UICC) [48]. All the patients underwent radical surgery. Patients with preoperative chemotherapy or radiotherapy treatment or with evidence of other malignancies were excluded. No patients received gene-targeted therapy during the follow-up period. Overall survival was defined as the time from the diagnosis until time of death from any cause. Cases lost to failure to follow-up and deaths caused by conditions other than lung cancer were regarded as censored data in the survival analysis. This study was approved by the medical ethics committee of Tongji Hospital and informed consent was obtained from all patients.

### Western blot analysis

The cell cultures and tissues were lysed in ice-cold RIPA lysis buffer (50 mMTris-HCl, 1% NP40, 0.1% SDS, 0.5% sodium deoxycholate, 0.02% sodium azide, 150 mM NaCl at pH 8.0) containing protease inhibitor cocktail (Roche, Switzerland). After the protein concentration was determined using a BCA Kit (Pierce, USA), 60 μg of proteins were separated on pre-casted 10% SDS-polyacrylamide gels and then electrotransferred onto PVDF membranes (Millipore, USA) in transfer buffer. The blots were blocked in 5% non-fat milk for two hours and incubated overnight at 4**°**C with primary antibodies (1:500 dilution). The blots were then incubated with horseradish peroxidase-conjugated secondary antibody at 1:5,000 dilution for one hour at 37°C. The signals were visualized using the enhanced chemiluminescence system (Pierce, USA). Protein expression was quantified by densitometry and normalized to β-actin expression using Alpha View software.

### Immunohistochemical staining

Antigen retrieval in the tissue sections was performed in boiling citrate buffer for 15 min. Peroxide blocking was conducted with 0.3% peroxide in absolute methanol. After the slides had been incubated overnight with primary antibodies (1:200 dilution) at 4°C and washed twice with PBS, they were then incubated with secondary antibody (Dako, Denmark) at 37°C for 30 min. After washing, the color reaction was developed with DAB work solution (Dako). The immunostaining results were assessed and scored independently by two pathologists as described previously [24].

### RNA isolation and qRT–PCR assays

Total RNAs from both fresh tissues and cell lines were extracted with Trizol reagent (Invitrogen, USA). MiRNAs from formalin-fixed and paraffin-embedded samples were extracted by using the RNeasy FFPE Kit (QIAGEN, Germany). The first-strand of complementary DNA was generated with a reverse-transcription system kit (TOYOBO, Japan). Stem-loop reverse transcription for mature hsa-miR-526b and U6 RNA was obtained from Ribobio Company (Guangzhou, China). U6 RNA was used as a miRNA internal control. qRT-PCR was performed with a standard SYBR-Green PCR kit (TOYOBO, Japan) according to a Step One system protocol (Applied Biosystems, Foster City, CA). The qRT-PCR reactions were performed in triplicate. Data analysis was performed using the 2^−ΔΔCt^ method.

### Cell culture and transfection

The A549 and PC-9 human NSCLC cell lines were obtained from the Center for Type Culture Collection of China and maintained in DMEM (Gibco, USA). Cells were supplemented with 10% fetal bovine serum (Gibco, USA). The A549 and PC-9 cells were transiently transfected with the RNAs or vector by using Lipofectamine 2000 (Invitrogen) following the manufacturer's protocol. The A549 cells were infected by GV320-Ku80 lentiviral vector and GV320 lentiviral vector according to manufacturer's protocol. At the indicated time points, the cells were harvested for use in the western blot analysis and cell proliferation assays. For stable transfections, 5 × 10^5^ cells per well were seeded in a six-well plate for 24 hours. Plasmid was delivered into the cells using Lipofectamine 2000 following the manufacturer's protocol. After culturing in medium containing 400 μg/ml of G418 (Calbiochem, Germany) for three weeks, the individual clones were isolated. The positive cell clones that stably expressed hsa-miR-526b were then identified using qRT-PCR. The clones stably expressing hsa-miR-526b were maintained in medium containing 250 μg/ml of G418 for further experiments.

### Luciferase reporter assay

To verify the precise target of miRNAs, the Dual-Luciferase Reporter Assay System (Promega, USA) was used. Briefly, 5 × 10^5^ NSCLC cells were seeded in 24-well plates. After 24 hours, the luciferase reporter plasmid was transfected or co-transfected with GV268-miR526b or its control vector using Lipofectamine 2000 (Invitrogen, USA). Firefly and renilla luciferase activities were measured at 48h post-transfection using the Dual-Glo Luciferase Assay System (Promega, USA). Firefly luciferase was normalized to renilla luciferase activity. All experiments were performed three times.

### Cell proliferation assay

Cells (5000 per well) were plated in 96-well plates and incubated at 37°C. Cell proliferation was assessed at indicated times after transfection using the Cell Counting Kit-8 (Dojindo, Japan) according to the manufacturer's instruction. Each assay was repeated independently three times.

### Analysis of clonogenicity *in vitro*

For plate colony formation analysis, 100 viable NSCLC cells were placed in 24-well plates 24 h after transfection and were maintained in complete medium for 2 weeks. Colonies were fixed with methanol and stained with 0.1% crystal violet in 20% methanol. Soft agar colony formation assay was performed as according to the methods described previously [24]. Briefly, 1,000 cells were equally divided into four wells in a 24-well plate in medium containing 0.3% noble agar and grown for 14–21 days. The number of colonies was determined by direct counting using an inverted microscope (Nikon, Japan).

### Flow cytometry for cell cycle and subG_1_ analysis

The cells were maintained in DMEM containing 10% serum for 24 hours, and were harvested and washed with PBS. Subsequently, 1 × 10^5^ cells were fixed with 70% ethanol. Immediately prior to the analysis, the cells were incubated with fresh propidium iodide containing RNase A for 30 min at 37°C. A total of 10^4^ cells were analyzed from each sample on a FACS Calibur flow cytometer (Becton Dickinson, USA). Cells in the subG_1_ phase have the least amount of DNA content in the cell cycle distribution, and are referred to as hypodiploid. The hypodiploid DNA content represents the fragmentation of DNA, indicating cell apoptosis [49].

### TUNEL assay

A TUNEL (terminal deoxynucleotidyl transferased UTP nick end labeling) assay, a commonly used method for detecting apoptotic programed cell death, was carried out by using the *in Situ* Cell Death Detection Kits (Roche Diagnostics, Switzerland) according to the manufacturer's instructions.

### Animal studies

BALB/c athymic nude mice (male, 4-week old and 16–20g) were purchased from Hubei Research Center of Laboratory Animal (Wuhan, China) and bred at pathogen-free conditions in Animal Center of Tongji Medical College. The hsa-miR-526b-expressing and NC-transfected clone cells were harvested and resuspended to 3 × 10^7^ cells/ml in PBS. We injected 3 × 10^6^ cells in a total volume of 100μl subcutaneously into the right flank of athymic nude mice (*n* = 9–10/clone). For the rescue experiment, the mice were randomly divided into six groups (*n* = 4/group). The initial four groups of mice, A549-NC (group 1), A549-miR-526#1 (group 2), A549-miR-526#1 transfected with GV320 (group 3), and A549-miR-526#1 transfected with GV320-Ku80 (group 4), received subcutaneous injections of cells into the flank. Two other groups received injections of A549-NC cells. Eight days were allowed for tumor development. After this period, Ku80 siRNA or control siRNA was directly injected into the implanted tumor at the dose of 1nmol (in 20 μl phosphate-buffered saline) per mouse every 4 days for a total of five times in each group. Tumor size was monitored every four days and measured using a caliper. The tumor volume was calculated by the formula V (cm^3^) = L × W^2^/2, where L represents the longest dimension and W the shortest dimension of the tumor. Twenty-four days after injection, the mice were sacrificed and their tumors removed. Tumor tissue fragments were fixed in 10% formalin and stored in −80**°**C at the same time. Expression of Ku80, p53, p21^Cip1/Waf1^ and ki67 in xenograft tumor tissues were detected by immunohistochemistry.

The A549-NC and A549-miR-526#1 clone cells (5 × 10^5^ cells in 50μl of PBS containing 25 ng of Matrigel; BD, USA) were injected into the left pleural cavities of 4-week old male athymic nude mice (*n* = 8 per group) to construct the orthotopic model according to the methods described previously [22]. The mice were sacrificed by overexposure to CO2 thirty days after implantation, at which time the lungs were removed and fixed in 10% formalin.

### Statistical analysis

All results represent the average from triplicate experiments are expressed as the mean ± standard deviation. The associations between categorical variables were assessed using the chi-square test or the Fisher exact test. Overall survival was calculated using the Kaplan–Meier method and log-rank tests. Analysis of variance (ANOVA) was performed to determine statistically significant differences between the groups. A value of *P* < 0.05 was considered statistically significant.

## SUPPLEMENTARY FIGURES


