# Supplementary material for: By downregulating Ku80, hsa-miR-526b suppresses non-small cell lung cancer
Source: Oncotarget. 2014 Dec 22;6(3):1462–77. doi: 10.18632/oncotarget.2808 (PMC4359307; doi:10.18632/oncotarget.2808)
Supplement: Supplementary file 1 [file oncotarget-06-1462-s001.pdf]

SUPPLEMENTARY FIGURES

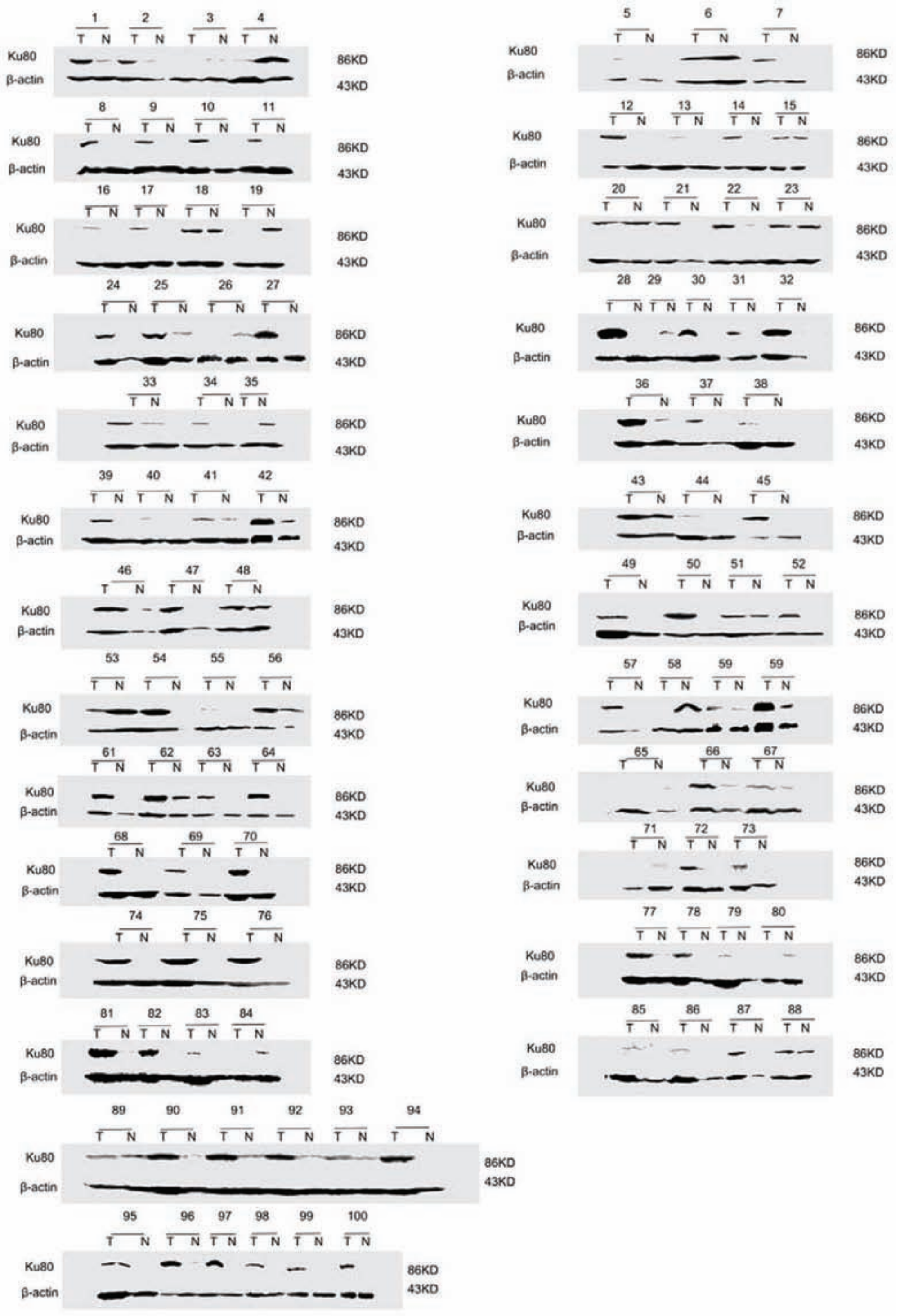

Supplementary Figure S1: Western blot showing Ku80 expression in NSCLC tissues (T) and corresponding adjacent lung tissues (N) from NSCLC patients. β-actin was included as an internal control.

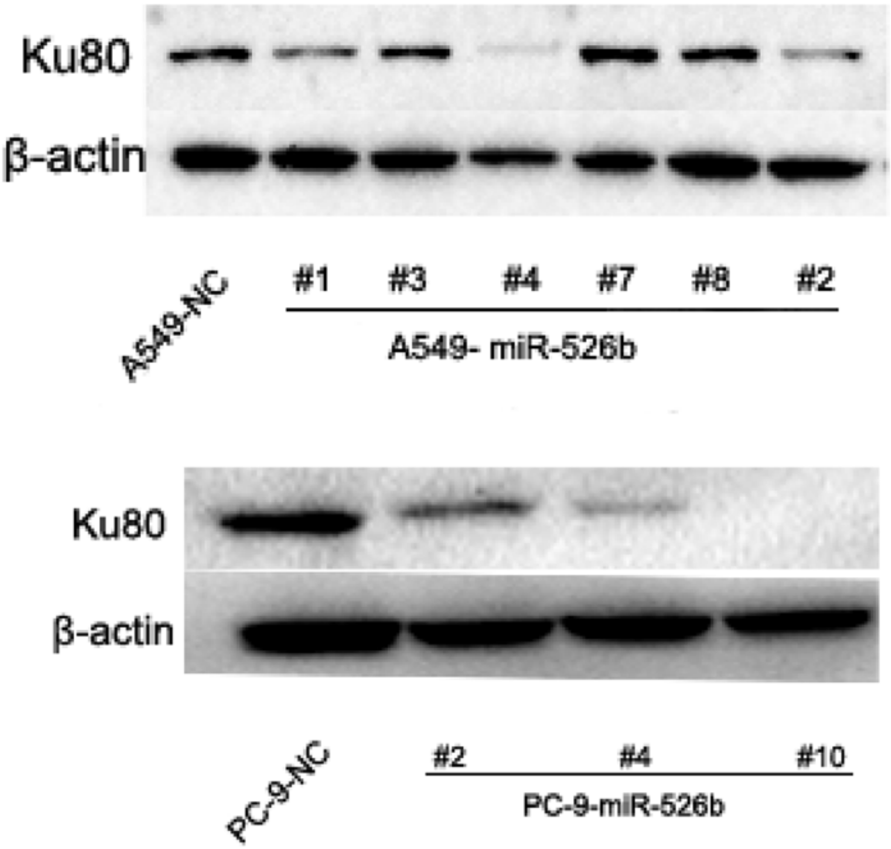

Supplementary Figure S2: Ku80 expression levels detected by western blot in two NSCLC cell lines (A549 and PC-9) stably transfected with hsa-miR-526b or hsa-miR-NC. β-actin was included as a loading control for each sample.

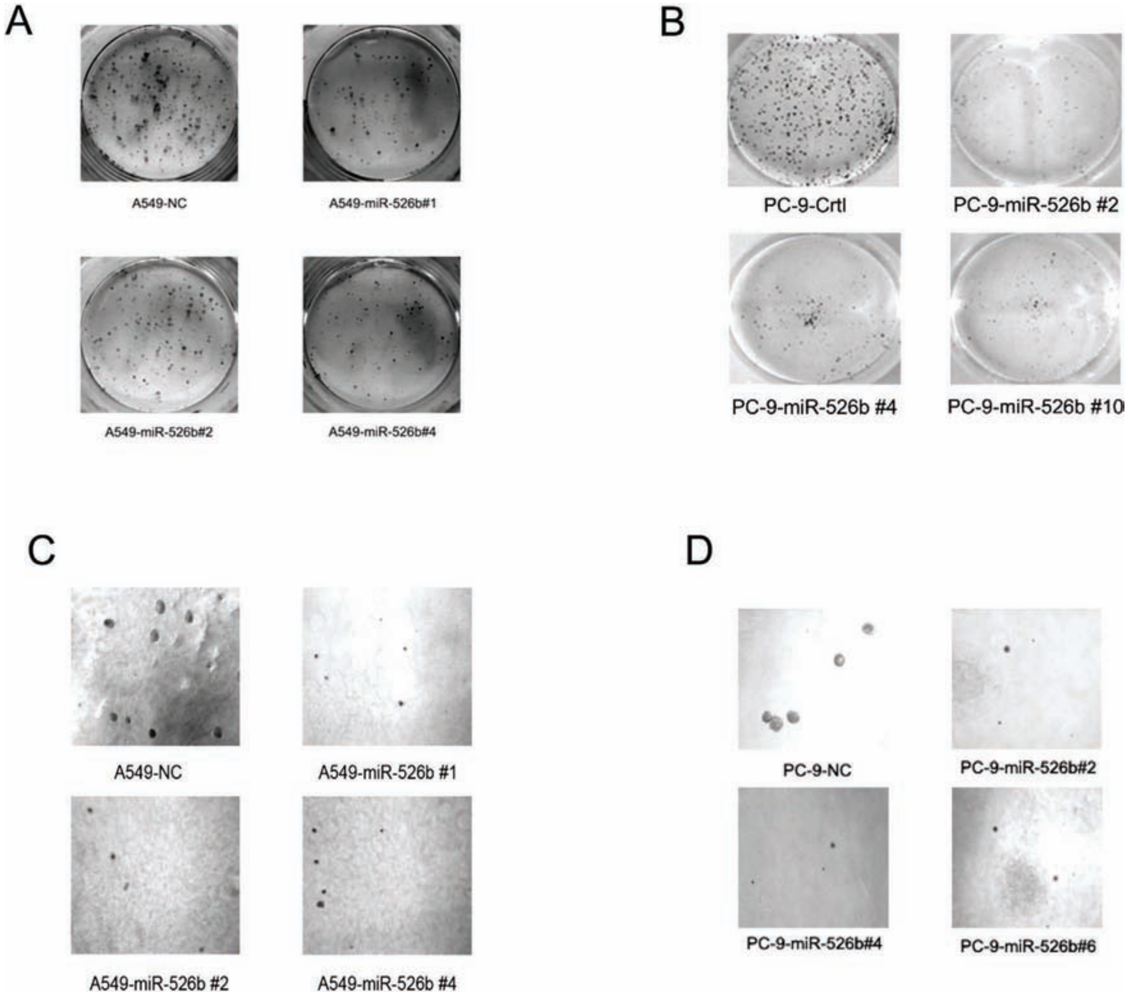

Supplementary Figure S3: Overexpression of miR-526b decreased cell clonogenicity both in A549 and PC-9 cells. Photographs showed representative results of plate colony formation using the indicated clones in A549 **(A)** and PC-9 **(B)** cells. Photographs show representative results of anchorage-independent growth in soft agar using the indicated clones in A549 **(C)** and PC-9 **(D)** cells.

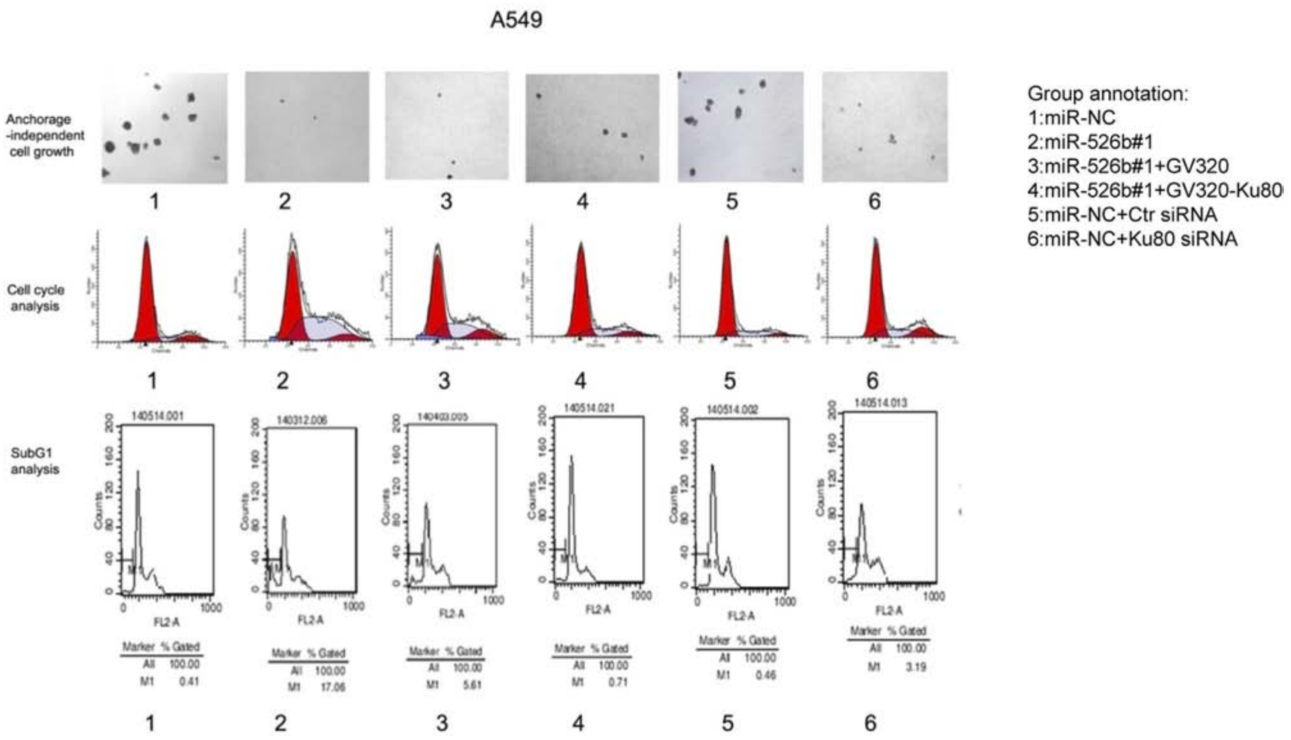

Supplementary Figure S4: The effect of Ku80 rescue or Ku80 silence on the anchorage-independent growth, cell-cycle distribution, and cell apoptosis rate in indicated cell groups. Anchorage-independent growth in soft agar **(a)** detected in the indicated cell groups 14 days after transfection, **(b)** cell cycle, and **(c)** cell apoptosis analysis determined in cell groups72 h after transfection.
